# Relationship between internet research data of oral neoplasms and public health programs in the European Union

**DOI:** 10.1186/s12903-021-02022-z

**Published:** 2021-12-17

**Authors:** Romain Lan, Fabrice Campana, Delphine Tardivo, Jean-Hugues Catherine, Jean-Noel Vergnes, Mehdi Hadj-Saïd

**Affiliations:** 1 APHM, CNRS, EFS, ADES, Timone Hospital, Oral Public Health Department, Aix Marseille Univ, Marseille, France; 2 APHM, INSERM, MMG, Timone Hospital, Oral Surgery Department, Aix Marseille Univ, Marseille, France; 3 APHM, CNRS, EFS, ADES, Timone Hospital, Oral Surgery Department, Aix Marseille Univ, 13005 Marseille, France; 4 Functional Unit of Epidemiology and Oral Public Health, Faculty of Odontology, Paul Sabatier University, Toulouse III, Toulouse, France; 5 Division of Oral Health and Society, Mc Gill University, Montreal, Canada; 6 Oral Surgery Department, APHM, CHU Timone, Marseille, France

**Keywords:** Oral cancer, Head and neck neoplasms, Health communication, Epidemiology, Mass screening, Prevention and control, Internet, Bibliometrics, Mass media

## Abstract

**Background:**

Tobacco and alcohol are the main risk factors for oral squamous cell carcinoma, the low survival rate of which is a public health problem. European-wide health policies (a prevention campaign, tobacco packaging) have been put in place to inform the population of the risks associated with consumption. Due to the increase in smoking among women, the incidence of this disease remains high. The identification of internet research data on the population could help to measure the impact of and better position these preventive measures. The objective was to analyze a potential temporal association between public health programs and interest in oral cancers on the internet in the European Union (EU).

**Methods:**

A search of data from Google ©, Wikipedia © and Twitter © users in 28 European countries relating to oral cancer between 2004 and 2019 was completed. Bibliometric analysis of press and scientific articles over the same period was also performed. The association between these data and the introduction of public health programs in Europe was studied.

**Results:**

There was a temporal association between changes in tobacco packaging and a significant increase in internet searches for oral cancer in seven countries. Unlike national policies and ad campaigns, the European awareness program Make Sense has had no influence on internet research. There was an asymmetric correlation in internet searches between publications on oral cancer from scientific articles or "traditional" media (weak association) and those from internet media such as Twitter © or Wikipedia © (strong association).

**Conclusion:**

Our work highlights seven areas around which oral cancer awareness in Europe could be refocused, such as a change in the communication of health warnings on cigarette packs, the establishment of a more explicit campaign name regarding oral cancer, the involvement of public figures and associations in initiatives to be organized at the local level and the strengthening of awareness of the dangers of tobacco in the development of oral cancer.

**Supplementary Information:**

The online version contains supplementary material available at 10.1186/s12903-021-02022-z.

## Background

### Oral cancers and their risk factors

A clear majority of oral cancers are attributable to tobacco and alcohol. The population should be informed about the potential consequences of their consumption [1, 2].

The increase in the consumption of tobacco among women and the evolution of sexual practices have led to an increase in oral and oropharynx cancers caused by human papillomavirus (HPV), respectively [3–5].

The low survival rate of oral cancers justifies effective prevention and screening.

### Preventing and raising awareness of oral cancers among the population of the European Union

By limiting the consumption of tobacco and alcohol and raising public awareness of their dangers, the worldwide prevalence of the disease could be reduced by 75% [6].

Prevention is also an economic issue. In Europe, the average annual cost of an oral cancer patient in 2012 was between €20.000 and €23.000 [7].

In the EU, smokers have been informed of the risks of tobacco consumption since the introduction of directive 2014/40/EU. Since 2016, this has forced the 28 member states to follow rules on the manufacturing, presentation and sale of tobacco and its derived products.

Since 2013, the Make Sense Campaign (MSC) has been raising awareness and providing information to the European population about head and neck cancers. Organized by the European Head & Neck Society (EHNS), the MSC involved 18 countries in 2018. In parallel, certain European countries have organized their own national campaigns [8].

### Evaluating the effects of health measures on the EU population

It would be tedious to survey a population as large as that of the EU to evaluate the large-scale impact of these measures.

Online research trends on the internet have been shown to reflect the changing trends of society over time. They show a marked increase in the number of internet searches during epidemics or for any other heightened interest in a disease [9, 10].

This has been demonstrated since the start of the COVID-19 pandemic [11].

The analysis of data obtained by such internet research would be effective for the study of illnesses, with precision comparable to normal epidemiological methods [12–14].

In 2016, Ayers et al. raised the possibility of using big data to quickly and cheaply evaluate the effects of awareness campaigns. They studied the effects of national no smoking days in the United States (Great American Smokeout) and showed that it led to a significant increase in the number of internet searches [15].

To be effective, an awareness campaign (screening or diagnosis) must attract the interest of the public, particularly in oncology, in which the objective is to make the population, particularly those at risk, aware of the usefulness of early detection [16]. Our goal, to assess the probable interest of the population, is to identify whether there is a temporal association between the implementation of an awareness campaign, or preventive measures, and the number of internet searches among the population. Due to the inherent limits of this kind of epidemiological research, this would not reflect a direct causal link between public health policies and interest shown by the populations but rather a potential temporal association, making it possible to enrich the reflection on the efficiency of awareness campaigns [17].

The objective was to analyze a potential temporal association between public health programs and interest in oral cancers on the internet in the EU.

We analyzed the search data related to oral cancers from Google Trends©, the bibliometric analysis of scientific articles on the topic, Wikipedia page consultations, and articles published in the press and on Twitter© and cross checked them against the data on the introduction of anti-tobacco public health care programs in the EU.

## Methods

### Working outline and inclusion criteria

This observational retrospective bibliometric analysis used search data collected from Google©, Wikipedia© and Twitter© users in the 28 EU countries between January 1^st^, 2004 and September 30th, 2018.

The data on press articles published and the bibliometric analysis of scientific articles during the same period were collected.

The temporal association between these results and the introduction of public health programs in the EU over the same period was studied.

On September 30, 2018, the countries included in this study had to be members of the EU, have an internet penetration rate (percentage of the population with internet access) of over 50%, have a Google© search engine usage rate of over 50% and participate in the MSC.

### Identification of public health programs related to oral cancers in the EU

The oral cancer risk factor prevention awareness campaigns of each EU country included were researched alongside public data from the World Health Organization (WHO) and the EHNS.

A search was carried out on the WHO website (http://www.euro.who.int) in the 'health topics' category. The health programs of each EU country were then identified using the 'Alcohol Use', 'Oral Health', 'Tobacco', 'Vaccines and Immunization' and 'Human Papillomavirus and Cervical Cancer' pages.

### Data collection

Data collection was standardized for each country.

A list of oral cancer clinical presentation key words that were compliant with the ICD-11 was created and translated into the EU’s 24 official languages. For countries with more than one official language, the key words in each official language were listed (Additional file 1: Annex 1) [18].

- Google Trends©

Search terms based on these keywords were entered into Google Trends© in the official language(s) of each included country to generate data linked to interest shown during the time period and in the geographical area studied.

The research was executed according to recommendations from Nuti et al. by entering each keyword as a 'search term' in the 'health' category [12].

Google Trends data do not provide an absolute value for interest in each search term. However, they do provide an index (relative search volume -RSV-) that refers to the number of searches completed for each term compared to the total number of searches completed on Google. This reported volume is scaled so that the maximum value on a given Google Trends search is 100.

Search terms that generated no data (RSV = 0) for a country were excluded from the analysis.

b) Wikipedia

The Wikipedia page view statistics for oral cancers in the languages of each of the included countries were collected from July 1, 2015 (date from which the statistics are publicly available), until September 30, 2018.

Data were collected using the same keywords as those previously used in the official language(s) of each country.

Pages whose view statistics were not available were not included.

iii) Twitter©

Public messages on Twitter© (tweets) about oral cancers between January 1, 2013 and September 30, 2018 were identified using a keyword search in the 24 official languages of the EU.

The start date was chosen empirically by the authors, considering that before 2013, this social network was not as widely used in the EU as in the period of 2013–2018. A preliminary search that found more than 100,000 tweets about oral cancers during this period confirmed the choice.

The keywords used were identical to those used for the collection of data on Google Trends© and Wikipedia.

The number of users posting tweets and the number of reactions ('retweets' and 'likes') were recorded.

iv) Europresse©

The Europresse database was used to evaluate media coverage of oral cancers.

A search of press articles was executed. The search area was limited to Europe between January 1, 2004, and September 30, 2018. The same search terms were used as those for Google Trends©, Wikipedia and Twitter©.

e) Bibliometric analysis

The Web of Science Core Collection and MEDLINE databases were used to complete a bibliometric analysis of scientific articles published between January 1, 2004, and December 31, 2018. The oral cancer key words were the same as those found in the MeSH (Medical Subject Headings), combined with the Boolean operator "OR": "mouth neoplasm", "mouth cancer", and "oral cancer".

### Graphical and statistical methods of analysis

Using the data generated by Google Trends©, Wikipedia, Twitter and Europresse©, descriptive statistics and scatterplots were created for each search term with adjusted polynomial trendlines [12, 19–21].

Linear regression demonstrated the evolutionary trend of the bibliometric analysis of scientific articles.

To observe the relationship between 1 (searches carried out on Google©, Wikipedia, Twitter, Europresse) and 2 (the introduction of health care programs), the student’s t test was implemented. Thus, one could compare the data linked to interest before and after the introduction of a health care program and assess the significance of its variations. Therefore, because the significance threshold is not impacted by a possible cumulative effect of the measurements over time, a Bonferroni correction was applied for each data collection.

Finally, Microsoft Excel© and MathWorks MATLAB© software were used to compare the search results from Google©, Wikipedia, Twitter, Europresse© and those from the bibliometric analysis using analysis of variance (ANOVA) and the Spearmann coefficient of linear correlation because the data not having a Gaussian distribution.

All statistical tests were used after verification of their application conditions.

## Results

### Public health programs

Each prevention measures we identified was about tobacco: the introduction of health warnings and shock images on cigarette packets in Belgium (2006), Spain (2011), France (2011), Romania (2008) and the United Kingdom (2008) and the enforcement of directive 2014/40/EU in all 28 EU countries.

Three awareness campaigns were analyzed: the MSC in Europe (every September since 2013), Mouth Cancer Awareness Day (MCAD) in Ireland (every September since 2010) and Mouth Cancer Action Month (MCAM) in the United Kingdom (every November since 1977).

### Google Trends©

Twenty EU countries were included, and eight countries (Cyprus, Greece, Hungary, Italy, Malta, Portugal, Romania and Sweden) were excluded due to a lack of usable data, particularly because of the impossibility of research on the four keywords for these countries.

In total, 43 searches in 17 languages for four keywords were completed: lip cancer (ten times), tongue cancer (thirteen times), gum cancer (six times) and mouth cancer (thirteen times).

**Fig. 1:**
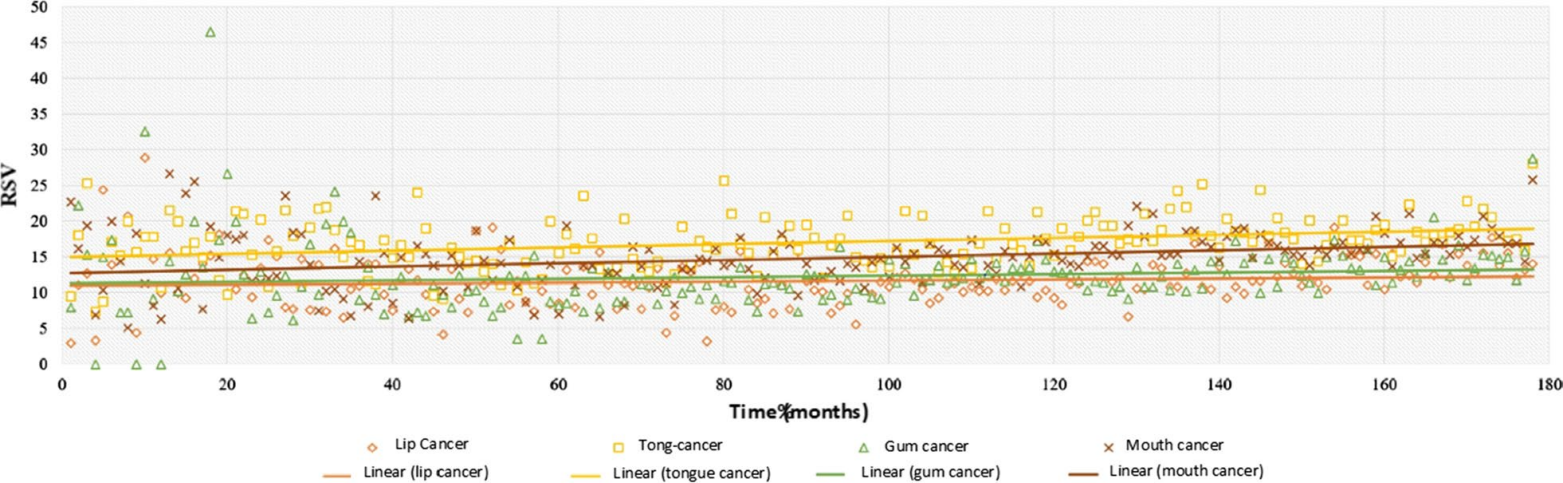
Evolution of RSV over time (2004–2018) for search terms in the countries included. The linear regression curves have the equation: lip cancer (y = 0.0074x + 11.007; R^2^ = 0.0112), tongue cancer (y = 0.0223x + 15.012; R^2^ = 0.1011), cancer gums (y = 0.0109x + 11.362; R^2^ = 0.0127) and oral cancer (y = 0.0225x + 12.795; R^2^ = 0.08

We noted a general increase in the popularity of the search terms over the period studied, with an average increase in interest of 8.1% (mouth cancer: 14.2%; lip cancer: 8.3%; gum cancer: 5.5%; tongue cancer: 4.5%). Figure 1 shows the scatter plots and linear regression curves for the four search terms across all countries included.

### Wikipedia

The statistics for Wikipedia page views connected to oral cancers were available in nine languages (Table 1).

**Table.1:** Visits of the Wikipedia pages on oral cancers in different languages, Descriptive statistics (July 2015–September 2018)

	Language	Page visits (2015–2018)	Monthly mean	Monthly median	Standard deviation	Variance	Range	Minimum	Maximum
1	English	966,625	24,785.25	24,120	3111	9,678,349.72	12,161	18,894	31,055
2	German	333,502	8551.33	8694	1851.90	3,429,543.54	3769	5206	12,739
3	Italian	147,616	3785.02	3767	981.37	963,085.39	3769	2145	5914
4	French	77,300	1982.05	1628	1170.44	1,369,946.89	6098	936	7034
5	Dutch	55,216	1415.79	1349	280.36	78,601.69	1140	838	1978
6	Polish	50,757	1301.46	1298	274.76	75,493.20	1295	799	2094
7	Portuguese	39,018	1000.46	1001	297.19	88,323.83	1380	477	1857
8	Slovenian	14,180	363.59	367	67.99	4622.62	307	257	564
9	Finnish	11,080	284.10	279	74.65	5572.30	316	167	483

**Fig. 2:**
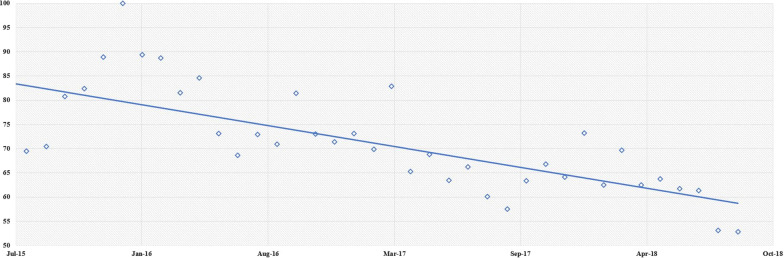
Evolution of the number of consultations of the 9 Wikipedia pages concerning oral cancers included according to time (2015–2018). The equation for the linear regression curve is: y =  − 0.0216x + 994.45; R^2^ = 0.5083

**Fig. 3:**
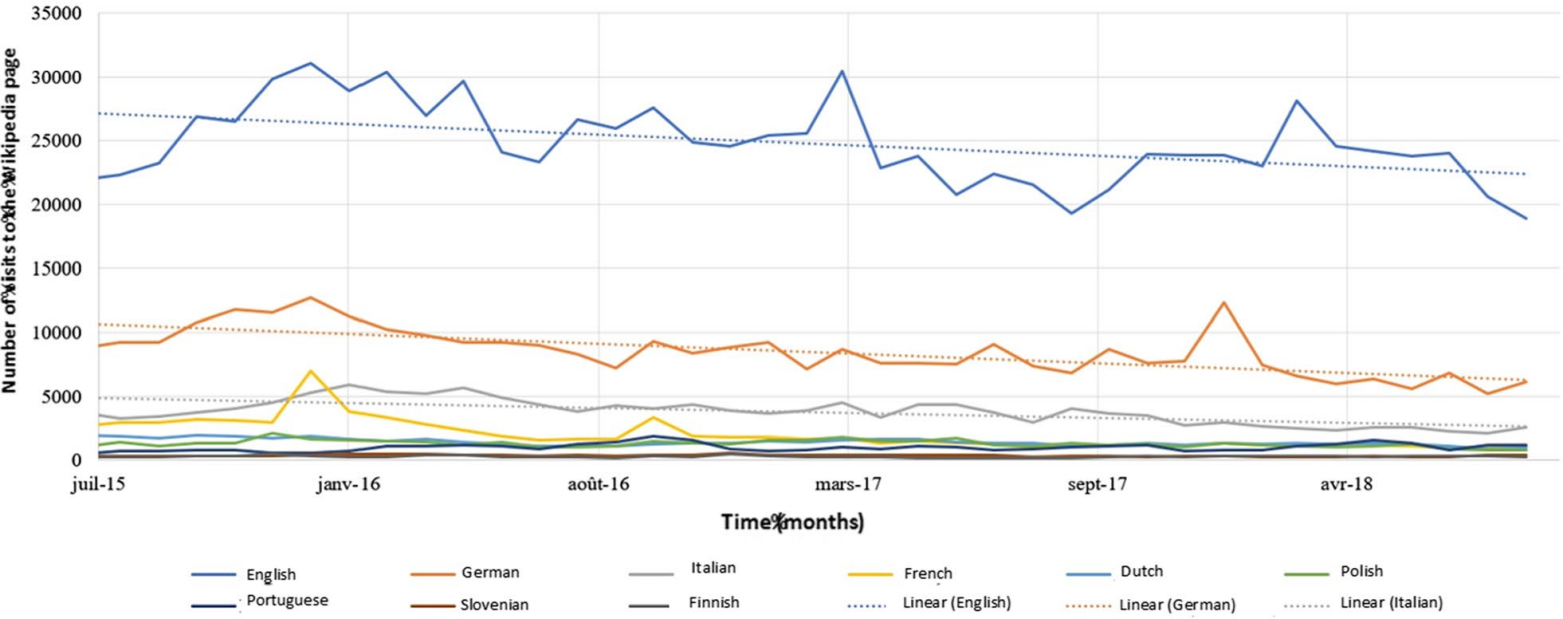
Evolution of the number of visits to Wikipedia pages over time (2015–2018) for search terms in the countries included. The linear regression curves have the following equation: English (y =  − 4.167x + 202,988; R^2^ = 0.2161), German (y =  − 3.8263x + 172,186; R^2^ = 0.5141), Italian (y =  − 1, 9807x + 88,489; R^2^ = 0.4906)

Figures 2 and 3 show the evolution of Wikipedia page views over the time period of 2015 to 2018. We noticed a slight decrease in interest in these pages (− 2.5%).

### Twitter©

125.595 tweets published by 49,168 users were documented in the 24 EU languages between January 1, 2013 and September 30, 2018. They generated 116,444 reactions (62,000 likes and 53,507 retweets).

On average, 1,820 tweets about oral cancers were published every month (median = 1427 standard deviation = 1268.81). The number of tweets decreased by 22.65% between 2013 and 2018.

Ninety-one percent of tweets about oral cancers were published in English. The 100 tweets with the most reactions were published using accounts with high numbers of followers and routinely relayed the oral cancer diagnosis of a public figure.

### Press articles

**Fig. 4:**
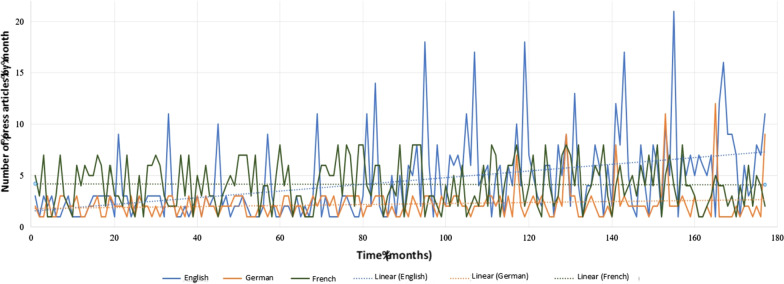
Evolution of the monthly number of articles published in the written press and the media in Europe as a function of time (2004–2018). The linear regression curves have the following equation: English (y = 0.0325x + 1.5496; R^2^ = 0.1793), French (y =  − 0.0006x + 4.2038; R^2^ = 0.0002), German ( y = 0.0047x + 1.7921; R^2^ = 0.0239)

Searches on Europresse revealed 787 articles in English, 735 in French and 392 in German (Fig. 4). Searches for articles in other languages did not return enough results to be useful.

On average, 4.44 articles about oral cancers in English (median = 3, standard deviation = 3.93), 4.15 in French (median = 4, standard deviation = 2.24) and 2.21 in German (median = 2, standard deviation = 1.57) appeared in the press every month between 2004 and 2018, with an overall increase of 12.2%.

### Bibliometric analysis

*A total of* 11,875 scientific articles about oral cancers were published worldwide between 2004 and 2018, with a yearly average of 789. The number of publications increased by 225% between 2004 and 2018.

Worldwide, the most prolific countries were the United States (3,693 articles; 31.1% of publications), China (1,710; 14.4%), Japan (1,561; 13.14%), Taiwan (1,486; 12.51%) and India (1,410; 11.8%).

A total of 33.7% of articles (3,998 articles) were published in the EU countries. The United Kingdom (6th; 1,068 articles; 8.99% of publications), Germany (8th; 557; 4.7%) and Italy (9th; 467; 3.93%) were the three most productive countries in the EU.

### Introduction of new public health care programs

The influence of the introduction of public health measures on the interest shown in oral cancers on Google©, Wikipedia and in the press is shown in Table 2.

**Table.2:** Influence of public health policies on Google © and Wikipedia searches for oral cancers and in press articles on the subject in Europe

	Country or language	Monthly mean	Mean before the 2014/40/EU directive	Mean before health warnings (countries having adopted them before the 2014/40/EU directive)	Mean before the introduction of a national campaign	Mean after the 2014/40/EU directive	Mean after health warnings (countries having adopted them before the 2014/40/EU directive)	Mean during MSC	Mean during a national campaign
*Google Trends© (RSV) 2004–2018*									
1	Germany	25.45	24.80	–	–	28.21 *p* = 0.175	–	26.38 *p* = 0.87	–
1bis	Germany (for the isolated term “Mundkrebs”)	23.21	21.68	–	–	**29.67** ***p* < 0.001**	–	28.3 *p* = 0.24	–
2	Austria	15.40	15.49	–	–	15.06 *p* = 0.842	–	12.21 *p* = 0.85	–
3	Bulgaria	9.35	9.19	–	–	9.99 *p* = 0.68	–	9.33 *p* = 1.00	–
3bis	Bulgaria (for the isolated term “Paк Ha Гъpдaтa”)	0.98	0.85	–	–	*1.5* *p* = *0.03*	–	1.66 *p* = 0.35	–
4	Croatia	11.50	12.03	–	–	9.26 *p* = 0.236	–	7.25 *p* = 0.86	–
5	Denmark	21.08	18.24	–	–	**33.15** ***p* < 0.001**	–	32.50 *p* = 0.17	–
6	Estonia	3.00	3.09	–	–	2.62 *p* = 0.808	–	0.00 *p* = 0.47	–
7	Finland	10.01	7.28	–	–	**21.53** ***p* < 0.001**	–	12.33 *p* = 0.69	–
8	Ireland	9.12	8.48	–	6.87	11.82 *p* = 0.18	–	**13.00** ***p* < 0.001**	10.4 p = 0.03
9	Latvia	3.60	3.24	–	–	5.10 *p* = 0.16	–	3.50 *p* = 0.97	–
10	Lithuania	4.66	3.92	–	–	7.79 *p* = 0.063	–	8.33 *p* = 0.41	–
11	Luxembourg	15.34	14.82	–	–	17.55 *p* = 0.283	–	15.44 *p* = 0.88	–
12	Netherlands	16.52	16.89	–	–	14.94 *p* = 0.527	–	10.50 *p* = 0.36	–
13	Poland	12.51	12.43	–	–	12.84 *p* = 0.764	–	8.00 *p* = 0.58	–
14	Czech Republic	4.16	4.15	–		4.20 *p* = 0.96	–	2.54 *p* = 0.69	
14bis	Czech Republic (for the isolated term “Rakovina Úst”)	0.37	0.27	–	–	0.79 *p* = 0.01	–	0.16 *p* = 0.64	–
15	Slovakia	10.07	9.48	–	–	12.59 *p* = 0.247	–	9.83 *p* = 0.97	–
16	Slovenia	1.63	1.60	–	–	1.79 *p* = 0.896	–	2.67 *p* = 0.75	–
*Countries with introduction of health warnings and shock images on cigarette packets before the enforcement of directive 2014/40/EU*									
17	Belgium	15.84	15.57	9.99	–	17.00 *p* = 0.741	16.73 *p* = 0.06	15.33 *p* = 0.88	–
17bis	Wallonia (keywords in French isolated)	27.5	26.95	12.25	–	29.82 *p* = 0.48	**29.87** ***p* = 0.02**	27.83 *p* = 0.97	–
18	Spain	30.66	30.17	27.57	–	32.74 *p* = 0.450	**33.43** ***p* = 0.03**	30.67 *p* = 1.00	–
19	France	16.03	15.32	14.51	–	**19.05** ***p* = 0.001**	**17.43** ***p* = 0.001**	17.28 *p* = 0.75	–
20	United Kingdom	24.82	23.79	26.1	24.82	**29.21** ***p* < 0.001**	24.35 *p* = 0.11	27.33 *p* = 0.35	*28.79* *p* = *0.02*
*Wikipedia (Number of page visits) 2015–2018*									
1	English	24,785	25,109	–	–	24,726 *p* = 0.78	–	21,836 *p* = 0.08	–
2	German	8551	10,269	–	–	42,874 *p* = 0.01	–	7352 *p* = 0.21	–
3	Italian	9785	3799	–	–	*3782* *p* = *0.02*	–	3612 *p* = 0.73	–
4	French	1982	2972	–	–	**1807** ***p* < 0.001**	–	1661 *p* = 0.59	–
5	Dutch	1416	1864	–	–	1285 *p* = 0.39	–	1211 *p* = 0.18	–
6	Polish	1301	1390	–	–	1285 *p* = 0.39	–	1096 *p* = 0.15	–
7	Portuguese	1000	682	–	–	**1058** ***p* < 0.001**	–	1067 *p* = 0.66	–
8	Slovenia	363	331	–	–	369 *p* = 0.2	–	341 *p* = 0.53	–
9	Finnish	284	321	–	–	277 *p* = 0.18	–	235 *p* = 0.21	–
*Europresse© (Number of articles) 2004–2018*									
1	English	4.44	3.86	–	–	6.3 *p* = 0.001	–	8.83 p = 0.007	**MCAD** **11.15** ***p* < 0.001** **MCAM** **9.17** ***p* < 0.001**
2	French	4.15	4.17	–	–	2.51 *p* = 0.34	–	**9.33** ***p* < 0.001**	–
3	German	2.21	2.02	–	–	3.78 *p* = 0.22	–	**5.33** ***p* < 0.001**	–

When one of the search terms studied isolated produced a significant result, it was highlighted (bis). When a research term studied produced an insignificant result due to Bonferroni's correction (to eliminate the cumulative effect of the different measures), it was noted in red italics

The countries for which introduction of health warnings and shock images on cigarette packets were introduced before the enforcement of directive 2014/40/EU have been placed separately, so as not to include any bias in the Bonferroni correction.

A significant increase in Google© searches followed the introduction of health warnings on cigarette packets in Spain (*P* = 0.03), France (*P* = 0.001) and in the French-speaking part of Belgium (*P* = 0.02). These countries introduced health warnings before the 2014/40/EU directive or the national or European prevention campaigns, and Bonferroni's correction could not be applied. We observed a significant increase in interest in oral cancers since the enforcement of directive 2014/40/EU in Denmark (*P* < 0.001), Finland (p < 0.001), France (*P* = 0.01) and the United Kingdom (*P* < 0.001). A significant increase in search terms corresponding to "mouth cancers" was also seen in Germany (p < 0.001), but not in Bulgaria (p = 0.003) and the Czech Republic (p = 0.01) due to the significance level with Bonferroni correction p = 0.05/39 = 0.00128).

The MSC had no influence on Google© searches, except in Ireland (*P* < 0.001).

Interest shown in oral cancers has increased significantly in Ireland since the month of September when the week of awareness raising (*P* < 0.001) coincided with the MSC.

However, the introduction of MCAD (*P* = 0.03) in Ireland or the MCAM in the United Kingdom (*P* = 0.02) did not significantly increase the search volume in these countries due to the potential cumulative effect of the introduction of health warnings on cigarette packs ahead of these campaigns (significance level with Bonferroni correction p = 0.0008).

In contrast, the data obtained for Wikipedia searches showed a significant decrease in the number of average monthly visits after the enforcement of directive 2014/40/EU on the French (*P* < 0.001) and Portuguese (*P* < 0.001) pages.

There was no significant temporal association between MSCs and the number of Wikipedia page visits concerning oral cancers.

**Fig. 5:**
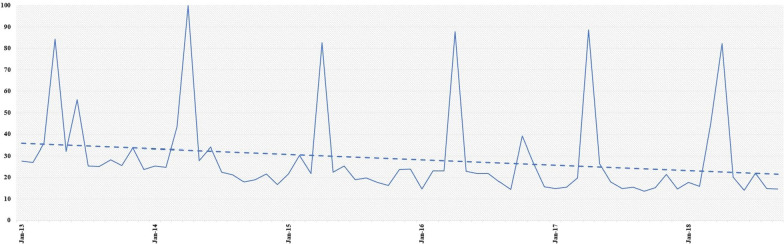
Evolution of the number of Tweets worldwide (2013–2018). The equation for the linear regression curve is: y =  − 0.4316x + 20,081; R^2^ = 0.04

On Twitter, the number of tweets increased significantly in April (*P* < 0.001), as shown in the regular peaks seen in Fig. 5. There was no change during the MSC (*P* = 0.13).

The study of the temporal association between the introduction of public health care programs and the publication of articles in the press showed a significant increase in the number of publications about oral cancers during each awareness campaign.

Several peaks of interest common to several databases were observed in September, 2010, January and October, 2016, March, 2017 and January, 2018.

The analysis of the relationship between the ANOVA results and the calculation of the Spearman correlation (Table 3) showed that associations and correlations existed between our results.

**Table.3:** Measurement of the Spearman correlation (ρ – rho) between the results obtained after ANOVA analysis

	Google Trends©	Wikipedia	Twitter©	Europresse	Bibliometrics
Google Trends©	–	ρ = 0.09 ***P*** < 0.001	ρ = 0.013 ***P*** < 0.001	ρ = 0.18 ***P*** < 0.001	ρ = 0.24 ***P*** < 0.001
Wikipedia	–	–	ρ = 0.09 ***P*** < 0.001	ρ = − 0.14 ***P*** < 0.001	Insufficient data
Twitter©	–	–	–	ρ = 0.89 ***P*** < 0.001	ρ = 0.98 ***P*** < 0.001
Europresse	–	–	–	–	ρ = 0.2 ***P*** < 0.001
Bibliometrics	–	–	–	–	–

There was no relationship between articles in the press and the number of Wikipedia page visits. Apart from this finding, the other results were positively associated. That is, the analyzed variables increased with one another.

A weak correlation was found between the publication of articles in the press about oral cancers and (1) the interest shown in them on Google© (0.11; *P* < 0.001) and (2) the publication of scientific articles (0.12; p < 0.001). We observed a weak correlation between the publication of scientific articles and interest shown in oral cancers on Google© (0.21; *P* < 0.001). Finally, a very strong correlation was found between the publication of scientific articles and (1) articles appearing in the press (0.8; *P* < 0.001) and (2) the number of tweets published (0.96; *P* < 0.001).

## Discussion

Our study shows the weak temporal association between the introduction of public health programs and the interest shown in oral cancers on the internet in most EU countries.

### Shock images and health warnings

These results reveal an increase in interest shown in oral cancers after the introduction of health warnings. It has already been shown that the type of explicit message associated with shock images impacts smokers [22–25].

Nevertheless, the use of shock images could have the opposite of the desired effect due to a saturation of overly directive messages [22–28].

These warnings could be accompanied by educational therapeutic medical information. In Canada and Australia, advice about how to quit smoking is printed on cigarette packets.

### European campaigns versus national campaigns

We have demonstrated the weak temporal association in interest shown in oral cancers on the internet during the MSC, excluding that shown by the press. An upturn in interest, although not significant, in Europe was observed in only Ireland and the United Kingdom, which are both countries that organized their own awareness campaigns. These are not organized uniquely by scholarly societies but on a smaller scale by dedicated foundations and associations that include patients in their organizational structure.

### The importance of social networks and celebrities

We highlighted the fact that the general population tends to follow the news rather than look for precise medical information.

Twitter posts that provoked the most reactions came from influential accounts. Celebrities can influence the wider public for a cause, at least for those paying attention to these "stars" [29–32].

Evans et al. described the "Angelina Jolie effect", noting a significant increase in breast cancer screening in the United States after the actress publicly announced her mastectomy in May, 2013 and called for more screening [33, 34].

We also linked an interview with a former baseball star (Jim Kelly) in March of 2017, which called for Americans to be tested during the Oral, Head & Neck Cancer Awareness campaign (OHNCA) to a spike in internet searches.

Awareness of oral cancers could be raised by the collaboration of celebrities who could inform their fan base about the consequences of their life choices.

### The influence of the names of prevention campaigns

The Wikipedia and Twitter© search tools include data from the United States in their English language search results since the algorithm does not allow for messages to be isolated or for searches by geographical area.

The United States organized an awareness campaign that appeared to generate an upturn in online interest. Oral cancers are clearly identified in the name of the campaign, such as those organized in Ireland and the United Kingdom, but in a way unlike the MSC.

A peak in interest in English databases in January, 2018, after the launch of the American screening campaign called, "Check Your Mouth™", confirms this assessment.

Figures 2 and 3 show a downward trend. This is the linear trend of the curve constructed from the monthly absolute values from Wikipedia and Twitter. It is decreasing. We have no specific explanation for this. It is very likely that this is because certain important values at the beginning of the period that we studied [2016 for Wikipedia (Fig. 1) and 2014 for Twitter (Fig. 2)] are responsible.

### Alcohol and HPV

Our study did not account for these risk factors due to the absence of a European awareness-raising policy specific to them.

Regarding risk factors that can influence the choice of keywords to better "understand" cancer, we found that the use of the Google Trends' "Related queries" function had no influence on the search selection strategy.

Our bibliometric analysis showed an increase in scientific interest in oral cancers.

This increase was particularly significant in January, 2016 following the publication of the article by Agalliu et al. that demonstrated the role of HPV-16 in the pathogenesis of oral cancers [35].

However, Syrjänen et al. already demonstrated this 35 years ago, even if the scientific impact was not as important as that observed today [36–38].

Increases in the number of online searches were also seen in September, 2010 and October, 2016 after Michael Douglas was asked about oral cancer. The increase in cancers attributed to HPV infection, although localized in the throat, may therefore explain these peaks of interest in the population.

The population should be informed of the risk of oropharynx cancers connected to contamination by HPV. Health care professionals, in addition to mouth head and neck specialists, should also be informed, particularly about the benefits of vaccination against HPV [39–41].

### Methodological tools

The objective of using several data sources was to increase the research’s relevance, although more exhaustively, by limiting the bias linked to the source effect of a single database. This type of research was inevitably limited in scope; the multiplication of sources, unlike conventional epidemiological surveys, makes it possible to better reflect on the desired effect [42, 43].

The use of Google and Wikipedia sources, which are common on the internet for information purposes, makes it possible to better identify the population's searches. The use of social networks such as Twitter, the most used network, is essential because it is as representative, if not currently more so, of trends and interest of the population on specific subjects, than any of the other networks [44, 45].

Finally, the bibliometric analysis and the press articles make it possible to add another level to the research and, thus, to refine the answer to our initial hypothesis by more precisely reflecting the interest of society in this issue of the fight against oral cancer [46].

### Limits

Unlike other databases used in this study, Google's© did not give the absolute number of page consultations related to oral cancers [12–14, 47–49].

Unlike a typical epidemiological investigation, populations and subpopulations could not be identified. The geographical area and inclusion period were vast and were not identical for all the databases analysed. It was not possible to know if the same internet users carried out multiple searches or performed searches from different devices.

The bibliometric period studied coincided with a general worldwide increase in the number of scientific publications, which is not, however, a guarantee of quality and scientific rigor [50–53].

Public health policies were not collected exhaustively, and the interest shown in oral cancers was dependent on an internet connection. The causality between these two factors could therefore be criticized and remain controversial. There are many potential confounding factors that could explain a correlation (as the COVID-19 pandemic has further demonstrated with rapid public action and a concomitantly increased number of connections) [54].

Thus, only a temporal association between public health programs and an interest in oral cancers on the internet in the EU can be presented [55].

The choice of keywords, which can sometimes be similar between sites despite different search preferences and can sometimes be unknown to the general public, is an inevitable bias in this type of research and a limitation that should be accounted for in the conclusions.

Similarly, there may have been a bias in the selection and interpretation of the results concerning social networks. It is not proven that all users of these platforms can be influenced by celebrities. These "star people" may therefore have less influence than supposed.

It was not possible to individually analyze the data sources used, such as Twitter accounts. There may therefore be a confounding factor in the analysis between the Twitter accounts of private individuals and those of public organizations that may echo awareness campaigns.

The concrete impact of policy measures on the number of screenings, consultations, diagnoses or waiting times was not considered in our work.

The temporal resolution (by week that carried over to the month) at which the data were extracted and analyzed could sometimes be underestimated. Research has shown that news-related spikes in search and social media typically return to baseline after approximately three days. However, this method makes it possible to not account for the parasitic results of the "daily" without losing those that would have appeared in a few days [56].

Conversely, awareness campaigns are vocal in the organization of screening sessions. Although the proof is limited, a visual examination during a screening campaign could reduce the oral cancer mortality rate among high-risk patients.

These campaigns therefore seem important in raising awareness for a scientifically well-documented condition that remains relatively unknown to the general public [57].

## Conclusion

We propose seven proposals that could reorient the awareness of oral cancer in Europe.

1. Modify the health warnings on cigarette packets to go beyond shock messages or images.
2. Encourage initiatives organized at a national level.
3. Involve celebrities and public figures in the promotion of awareness campaigns.
4. Encourage greater involvement of associations and foundations in the organization of the MSC.
5. Rename the MSC since it does not currently evoke a medical awareness campaign. In particular, the general public remains unaware of oral cancers.
6. Rename "head and neck cancers" as "mouth, head and neck cancers".
7. Implement an awareness-raising policy of the dangers of tobacco and other risk factors.

## Supplementary Information

**Additional file 1: Annex 1**. Search terms used. Descriptive statistics (European Union, 2004–2018).

## Data Availability

The datasets used and/or analysed during the current study are available from the corresponding author on reasonable request.
