# Supplementary material for: Relationship between internet research data of oral neoplasms and public health programs in the European Union
Source: BMC Oral Health. 2021 Dec 17;21:648. doi: 10.1186/s12903-021-02022-z (PMC8679572; doi:10.1186/s12903-021-02022-z)
Supplement: Supplementary file 1 — Additional file 1: Annex 1. Search terms used. Descriptive statistics (European Union, 2004–2018). [file 12903_2021_2022_MOESM1_ESM.docx]

|  | Contry | Official language(s) | Keywords | Mean RSV | Median RSV | Standard deviation | Variance | Mode | Minimum | Maximum |
| --- | --- | --- | --- | --- | --- | --- | --- | --- | --- | --- |
| **1** | Germany | German | Lippenkrebs  Zungenkrebs  Zahnfleischkrebs  Mundkrebs | 11,3  65,9  1,3  25,4 | 10  67  0  24 | 6,6  13,9  2,3  10,5 | 43,2  192,2  5,3  110,1 | 9  71  0  24 | 0  28  0  0 | 35  100  17  73 |
| **2** | Austria | German | Lippenkrebs  Zungenkrebs  Zahnfleischkrebs  Mundkrebs | 5,5  31  0  9,7 | 4  29,5  0  7 | 8,5  17,5  0  14,2 | 72,7  307,2  0  202,4 | 0  0  0  0 | 0  0  0  0 | 70  100  0  97 |
| **3** | Belgium | German  French  Dutch | Lippenkrebs  Zungenkrebs  Zahnfleischkrebs  Mundkrebs  Cancer de la lèvre  Cancer de la langue  Cancer des gencives  Cancer de la bouche  Lipkanker  Kanker van de tong  Tandvleeskanker  Kanker van de mond | 0  0  0  0  0  33,2  0  21,8  4,2  0  0  0 | 0  0  0  0  0  33  0  17  0  0  0  0 | 0  0  0  0  0  24  0  21,8  11,8  0  0  0 | 0  0  0  0  0  578,2  0  471,6  139  0  0  0 | 0  0  0  0  0  0  0  0  0  0  0  0 | 0  0  0  0  0  0  0  0  0  0  0  0 | 0  0  0  0  0  0  0  0  100  0  0  0 |
| **4** | Bulgaria | Bulgarian | Рак на устните  Рак на езика  Рак на гърдата  Рак на устата | 0  0  17,7  1 | 0  0  15  0 | 0  0  14  1,8 | 0  0  196,2  3,2 | 0  0  0  0 | 0  0  0  0 | 0  0  100  10 |
| **5** | Cyrpus | Greek  Turkish | Ο καρκίνος των χειλιών  Καρκίνος της γλώσσας  Καρκίνος καρκίνου  Καρκίνος του στόματος  Dudak kanseri  Dil kanseri  Sakız kanseri  Ağız kanseri | 0  0  0  0  0  0  0  0 | 0  0  0  0  0  0  0  0 | 0  0  0  0  0  0  0  0 | 0  0  0  0  0  0  0  0 | 0  0  0  0  0  0  0  0 | 0  0  0  0  0  0  0  0 | 0  0  0  0  0  0  0  0 |
| **6** | Croatia | Croatian | Rak dojke  Rak jezika  Rak gume  Rak usta | 22  0,8  0  0 | 18  0  0  0 | 16,7  1,8  0  0 | 277,6  3,1  0  0 | 15  0  0  0 | 0  0  0  0 | 100  13  0  0 |
| **7** | Denmark | Danish | Læft kræft  Tunge kræft  Gum cancer  Kræft i munden | 0  0  0  21 | 0  0  0  21 | 0  0  0  20,2 | 0  0  0  408,4 | 0  0  0  0 | 0  0  0  0 | 0  0  0  100 |
| **8** | Spain | Spanish | Cáncer de labio  Cáncer de la lengua  Cáncer de encías  Cancer de la boca | 0  0  0  30,6 | 0  0  0  31 | 0  0  0  17,7 | 0  0  0  313,9 | 0  0  0  0 | 0  0  0  0 | 0  0  0  100 |
| **9** | Estonia | Estonian | Huultevähk  Keelevähk  Igemevähk  Suu vähk | 0  3  0  0 | 0  0  0  0 | 0  10,1  0  0 | 0  103,2  0  0 | 0  0  0  0 | 0  0  0  0 | 0  100  0  0 |
| **10** | Finland | Finnish  Swedish | Huulirasva  Kielen syöpä  Gum syöpä  Suusyöpä  Lip cancer  Tungans cancer  Gum cancer  Munkreft | 10  0  0  0  0  0  0  0 | 4  0  0  0  0  0  0  0 | 14  0  0  0  0  0  0  0 | 197,7  0  0  0  0  0  0  0 | 0  0  0  0  0  0  0  0 | 0  0  0  0  0  0  0  0 | 100  0  0  0  0  0  0  0 |
| **11** | France | French | Cancer de la lèvre  Cancer de la langue  Cancer des gencives  Cancer de la bouche | 0  31,3  1,7  15 | 0  31  1  15 | 0  10,3  2,3  5,3 | 0  107  5,4  28,2 | 0  31  2  15 | 0  0  0  0 | 0  100  18  42 |
| **12** | Greece | Greek | Ο καρκίνος των χειλιών  Καρκίνος της γλώσσας  Καρκίνος καρκίνου  Καρκίνος του στόματος | 0  0  0  0 | 0  0  0  0 | 0  0  0  0 | 0  0  0  0 | 0  0  0  0 | 0  0  0  0 | 0  0  0  0 |
| **13** | Hungary | Hungarian | Liprák  A nyelv rákja  Gumrák  A száj rákja | 0  0  0  0 | 0  0  0  0 | 0  0  0  0 | 0  0  0  0 | 0  0  0  0 | 0  0  0  0 | 0  0  0  0 |
| **14** | Ireland | Irish  English | Ailse Lip  Ailse an teanga  Ailse gum  Ailse an bhéil  Lip cancer  Tongue cancer  Gum cancer  Mouth cancer | 0  0  0  0  9,1  0  0  0 | 0  0  0  0  8  0  0  0 | 0  0  0  0  13,1  0  0  0 | 0  0  0  0  172,5  0  0  0 | 0  0  0  0  0  0  0  0 | 0  0  0  0  0  0  0  0 | 0  0  0  0  100  0  0  0 |
| **15** | Italiy | Italian | Cancro al labbro  Cancro della lingua  Cancro alla gomma  Cancro della bocca | 0  0  0  0 | 0  0  0  0 | 0  0  0  0 | 0  0  0  0 | 0  0  0  0 | 0  0  0  0 | 0  0  0  0 |
| **16** | Latvia | Latvian | Lūpu vēzis  Mēles vēzis  Gumijas vēzis  Mutes vēzis | 0  1,7  0  5,4 | 0  0  0  0,9 | 0  3,9  0  12,6 | 0  15,4  0  158,8 | 0  0  0  0 | 0  0  0  0 | 0  23  0  100 |
| **17** | Lithuania | Lituanian | Lūpos vėžys  Liežuvio vėžys  Gumos vėžys  Burnos vėžys | 0  0  0  4,6 | 0  0  0  0 | 0  0  0  10,9 | 0  0  0  119 | 0  0  0  0 | 0  0  0  0 | 0  0  0  100 |
| **18** | Luxembourg | Luxembourgish  German  French | Lipkresskriibs  Kriibs vun der Zong  Gumskrankheeten  Kriibs vum Mound  Lippenkrebs  Zungenkrebs  Zahnfleischkrebs  Mundkrebs  Cancer de la lèvre  Cancer de la langue  Cancer des gencives  Cancer de la bouche | 11,3  65,9  1,3  25,4  0  5,2  0  0  0  0  0  0 | 10  67  0  24  0  0  0  0  0  0  0  0 | 6,6  13,9  2,3  10,5  0  12,4  0  0  0  0  0  0 | 43,2  192,2  5,3  110,1  0  153,6  0  0  0  0  0  0 | 9  71  0  24  0  0  0  0  0  0  0  0 | 0  28  0  0  0  0  0  0  0  0  0  0 | 35  100  17  73  0  100  0  0  0  0  0  0 |
| **19** | Malta | Maltese  English | Kanċer tal-lip  Kanċer tal-ilsien  Kanċer tal-gomma  Kanċer tal-ħalq  Lip cancer  Tongue cancer  Gum cancer  Mouth cancer | 0  0  0  0  0  0  0  0 | 0  0  0  0  0  0  0  0 | 0  0  0  0  0  0  0  0 | 0  0  0  0  0  0  0  0 | 0  0  0  0  0  0  0  0 | 0  0  0  0  0  0  0  0 | 0  0  0  0  0  0  0  0 |
| **20** | Netherlands | Dutch | Lipkanker  Kanker van de tong  Tandvleeskanker  Kanker van de mond | 16,5  0  0  0 | 14  0  0  0 | 16  0  0  0 | 258,8  0  0  0 | 0  0  0  0 | 0  0  0  0 | 100  0  0  0 |
| **21** | Poland | Polish | Rak warg  Rak języka  Rak gumy  Nowotwory szczęki | 3,4  15,6  0  18,5 | 3  14  0  18 | 4,5  12,9  0  12 | 20,7  166,3  0  146 | 0  0  0  0 | 0  0  0  0 | 33  99  0  100 |
| **22** | Portugal | Portuguese | Câncer de lábio  Câncer da Língua  Câncer de goma  Câncer da boca | 0  0  0  0 | 0  0  0  0 | 0  0  0  0 | 0  0  0  0 | 0  0  0  0 | 0  0  0  0 | 0  0  0  0 |
| **23** | Czech Republic | Czech | Rakovina rtů  Rakovina jazyka  Rakovina žaludku  Rakovina úst | 0  4,1  8  0,4 | 0  3  7  0 | 0  6  10,7  1,1 | 0  36,8  115,5  1,2 | 0  0  0  0 | 0  0  0  0 | 0  42  100  8 |
| **24** | Roumania | Roumanian | Cancerul buzelor  Racul limbii  Gum cancer  Cancerul gurii | 0  0  0  0 | 0  0  0  0 | 0  0  0  0 | 0  0  0  0 | 0  0  0  0 | 0  0  0  0 | 0  0  0  0 |
| **25** | UK | English | Lip cancer  Tongue cancer  Gum cancer  Mouth cancer | 22,7  17,3  32,9  26,7 | 22,5  16  33  25 | 10,8  9,4  14,1  11,8 | 117,3  89,6  199,2  139,9 | 22  14  36  26 | 0  0  0  0 | 75  65  100  90 |
| **26** | Slovakia | Slovak | Rakovina pier  Rakovina jazyka  Rakovina húb  Rakovina úst | 0  10,1  0  0 | 0  7  0  0 | 0  14  0  0 | 0  197,4  0  0 | 0  0  0  0 | 0  0  0  0 | 0  100  0  0 |
| **27** | Slovenia | Slovenian | Rak za ustnice  Rak jezika  Rak gumija  Rak ust | 0  1,6  0  0 | 0  0  0  0 | 0  7,8  0  0 | 0  61,5  0  0 | 0  0  0  0 | 0  0  0  0 | 0  100  0  0 |
| **28** | Sweden | Swedish | Lip cancer  Tungans cancer  Gum cancer  Munkreft | 0  0  0  0 | 0  0  0  0 | 0  0  0  0 | 0  0  0  0 | 0  0  0  0 | 0  0  0  0 | 0  0  0  0 |
|  | **Total contries included (20)** | Germany, Austria, Belgium, Bulgaria, Croatia, Denmark, Spain, Estonia, Finland, France, Ireland, Latvia, Lithuania, Luxembourg, Netherlands, Poland, Czech Republic, United Kingdom, Slovakia and Slovenia. | Keyword 1 (translation of "lip cancer")  Keyword 2 (translation of "cancer of the tongue")  Keyword 3 (translation of "gum cancer")  Keyword 4 (translation of "mouth/oral cancer") | 11,6  17  12,3  14,8 | 9  8  6  12 | 13,8  22  15,6  16,5 | 191,3  488,1  245,2  272 | 0  0  0  0 | 0  0  0  0 | 100  100  100  100 |

**Annex 1**. Search terms used. Descriptive statistics (European Union, 2004-2018).
